# Radial Nerve Palsy as an Iatrogenic Complication of Shoulder Replacement Surgery With Significant Bone Loss of the Humerus Resembling Gorham–Stout Disease: Case Report and Review of the Literature

**DOI:** 10.1155/crnm/9969463

**Published:** 2025-06-19

**Authors:** Lisa B. E. Shields, Vasudeva G. Iyer, Yi Ping Zhang, Christopher B. Shields

**Affiliations:** ^1^Norton Neuroscience Institute, Norton Healthcare, Louisville 40202, Kentucky, USA; ^2^Neurodiagnostic Center of Louisville, Louisville 40245, Kentucky, USA

**Keywords:** electrodiagnostic studies, Gorham–Stout disease, massive osteolysis, nerve injury, neurology, ultrasound studies, vanishing bone disease

## Abstract

Gorham–Stout disease (GSD), also known as vanishing bone disease or massive osteolysis, is a rare entity characterized by destruction of the osseous matrix and proliferation of vascular structures resulting in bone resorption. While neurological complications such as cerebrospinal rhinorrhea secondary to cranial involvement and paraplegia from spinal involvement have been reported, peripheral nerve complications are not known. We describe a case of radial nerve palsy that was an iatrogenic complication of shoulder replacement surgery with bone loss of the humerus resembling GSD. A 71-year-old male with a history of left total shoulder arthroplasty followed by a revision reverse total shoulder arthroplasty noted a “bone protruding” and pain in the left upper arm 12 years later. X-rays showed that the proximal portion of the humerus was not detectable. CT scan of the left upper extremity revealed loosening of the humeral component with prominent osteolysis most pronounced around the distal stem. The patient underwent a revision of the reverse total shoulder arthroplasty with replacement of the humeral head and shaft. He experienced numbness, pain, and weakness of the left shoulder and arm with wrist drop postoperatively. Physical exam revealed marked weakness of the dorsiflexors of the wrist and digits, wasting and weakness of the brachioradialis muscle, and loss of pinprick sensation of the superficial radial nerve distribution. Needle EMG showed denervation changes in the extensor digitorum communis, brachioradialis, and extensor carpi radialis longus muscles. An ultrasound (US) study showed enlargement of the left radial nerve at the spiral groove. The EDX and US findings suggested a left radial nerve palsy at the spiral groove. There were minimal EMG abnormalities in the deltoid and triceps muscles suggesting additional involvement of the posterior cord of the brachial plexus. This case illustrates the potential for iatrogenic radial nerve palsy following shoulder replacement surgery with significant bone loss of the humerus resembling GSD.

## 1. Introduction

In 1838, Jackson initially described a case of “a boneless arm” where the humerus underwent massive osteolysis until it disappeared within 11 years [1]. Gorham et al. reported two cases of massive osteolysis in 1954 [2], and Gorham and Stout subsequently reviewed 24 patients with this condition in 1955 [3]. The Gorham–Stout disease (GSD), also known as vanishing or phantom bone disease or massive osteolysis, is marked by progressive destruction and resorption of bone due to abnormal proliferation of vascular or lymphatic vessels [4–7]. Fibrous tissue replaces bone marrow, and trabeculae become thinner [4]. Bone-related swelling, pain, restricted range of motion, osseous deformity, and weakness are the classic features of this condition, primarily involving the maxillofacial area, shoulder, proximal humerus, pelvic girdle, and skull [4–12]. Most cases involve a single bone; multiple sites with GSD are rare [13]. Symptoms may develop spontaneously or be caused by a pathologic fracture that may be atraumatic or occur following minor trauma [5, 6]. GSD may extend across the joint line to affect an entire anatomical area [9]. This condition is often self-limited with symptoms often stabilizing with pharmacologic treatment [4, 5]. Severe neurological complications may greatly increase the risk of morbidity and mortality and may include cerebrospinal fluid leakage often complicated by meningitis (if the lesion involves the skull base or spine) or paraplegia (if there is spinal cord involvement) [5–11]. Intracranial hypotension and less often intracranial hypertension and acquired Chiari malformation have been reported [14]. Pleural complications include pleural effusion and chylothorax. This condition is usually diagnosed in patients less than 40 years of age, with an average age of 25 years [5–7]. In Angelini et al.'s systematic review of GSD between 1955 and 2021, over 350 cases have been reported in 270 articles [5].

The etiology of GSD is unclear. However, three etiopathological criteria have been proposed as being integral to GSD, including (1) increased osteoclast population, (2) angiogenesis and lymphangiogenesis, and (3) disruption of osteoblast function [4, 5]. The enhanced vascularization alters the osteoblast–osteoclast equilibrium which encourages bone resorption [4]. It has been postulated that massive osteolysis is secondary to angiomatosis involving proliferation of vascular channels (blood, lymphatic, capillary, or cavernous) that causes active hyperemia resulting in bone absorption [8, 9].

We present an unusual case of iatrogenic injury of the radial nerve that occurred in the intraoperative period for shoulder replacement surgery with significant bone loss of the humerus resembling GSD. The diagnostic tests, differential diagnosis, and treatment of GSD are discussed. We also highlight the EDX and US findings of radial neuropathy that may develop following shoulder arthroplasty.

## 2. Case Description

### 2.1. History, Radiological Findings, and Physical Examination

**Figure 1:**
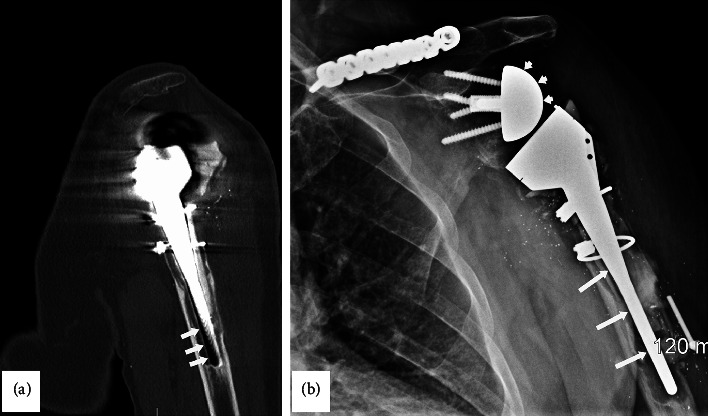
(a) CT scan of the left shoulder that shows the metal prosthesis of the upper half of the humerus. Note erosion of the bone surrounding the lower end of the prosthesis (arrows). (b) AP radiograph of the left shoulder demonstrating the arm and shoulder with bone loss surrounding the lower end of the prosthesis (arrows).

A 71-year-old male sustained an injury to the left shoulder 23 years prior to presentation at our Neurodiagnostic Center, at which time he underwent a left total shoulder arthroplasty. Eleven years before presentation, he had a reverse total shoulder arthroplasty. Six months prior to presentation, a CT scan of the left shoulder showed a metal prosthesis of the upper half of the humerus with erosion of the bone surrounding the lower end of the prosthesis (Figure 1(a)). A radiograph of the left shoulder demonstrated the arm and shoulder with bone loss surrounding the lower end of the prosthesis (Figure 1(b)). The patient underwent a revision of the reverse total shoulder arthroplasty which consisted of replacing the left humeral head and shaft. There was severe bone loss and loosening of the components as well as a broken router bit. The procedure involved an augmented base plate of the glenoid with bone grafting of the glenoid, segmental replacement of the humerus, hardware removal including the broken router bit and extensive scar tissue release. The shoulder itself was grossly unstable, and the humeral component was loose. Broken cement was noted throughout the subdeltoid space and more distally. There was a significant amount of bone loss on the humeral side with complete loss of the rotator cuff and most of the soft tissue attachments. There was also a significant amount of bone loss on the glenoid side with erosion.

The patient complained of numbness, pain, and weakness of the left shoulder, upper arm, and forearm postoperatively with an inability to dorsiflex the wrist and fingers at the MP joints. The patient subsequently underwent two additional left revision reverse total shoulder arthroplasties over the next 3 months to stabilize the head of the humerus. The left shoulder and forearm pain as well as weakness of left upper extremity persisted after these surgeries.

**Figure 2:**
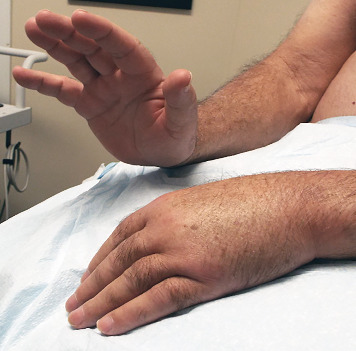
Left wrist drop due to weakness of the extensor muscles of the wrist. The brachioradialis muscle was also weak.

The patient was subsequently referred to our Neurodiagnostic Center for left wrist drop. He reported a 6-month history of a “bone sticking out under the skin” in the left upper arm accompanied by pain. Physical exam revealed marked weakness of the left dorsiflexors at the wrist as well as digits (Figure 2). Wasting and weakness of the brachioradialis muscle was also noted. Loss of pinprick sensation in the distribution of the left superficial radial nerve were detected. There was also fixity of the left shoulder joint.

### 2.2. EDX and Ultrasound Studies

**Figure 3:**
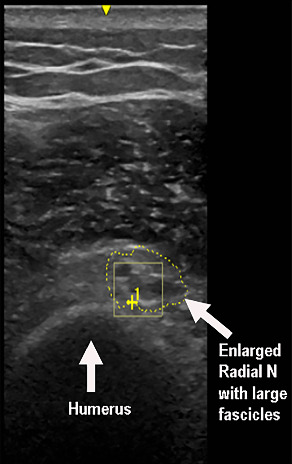
Ultrasound showed enlargement of the radial nerve with large fascicles at the spiral groove.

Stimulation of the left radial nerve did not evoke compound muscle action potentials over the extensor digitorum communis (EDC) or extensor indicis muscles. Superficial radial nerve stimulation did not evoke sensory nerve action potentials (SNAPs). Needle EMG showed denervation changes in the EDC, brachioradialis, and extensor carpi radialis longus muscles; one to two small polyphasic units were recruited in the brachioradialis muscle. The triceps muscle showed reinnervation changes with almost normal motor unit recruitment, while the deltoid muscle demonstrated decreased motor units with large polyphasic units with no fibrillations. An US study showed enlargement of the left radial nerve at the spiral groove (Figure 3).

These findings suggested a left radial nerve axonal injury distal to the innervation of the triceps muscle, most likely at the spiral groove with only minimal reinnervation limited to the brachioradialis muscle. It is likely that the patient sustained an intraoperative injury to the radial nerve at the level of the spiral groove during the reverse total shoulder arthroplasty revision when the humeral head and shaft were replaced. Based on the EMG patterns of the triceps and deltoid muscles, there was also involvement of the posterior cord likely due to a stretch injury.

### 2.3. Follow-Up

**Figure 4:**
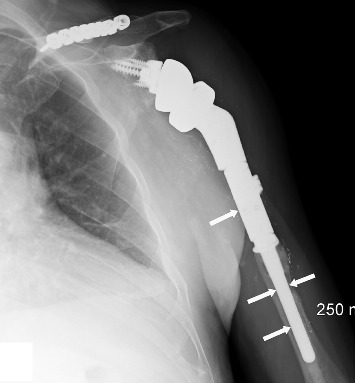
AP view of the shoulder and upper arm showing excellent placement and alignment of the humeral prosthesis following the reverse total shoulder arthroplasty with no evidence of hardware (arrows) loosening.

Four months after the EDX studies, the patient underwent a tendon transfer for the left radial nerve palsy, consisting of transfer of the left palmaris longus to the extensor pollicis longus and left flexor carpi radialis to the finger extensors. Left finger and thumb function had improved by 3 weeks postoperatively. A radiograph of the left shoulder and upper arm showed excellent placement and alignment of the humeral prosthesis following the reverse total shoulder arthroplasty with no evidence of hardware loosening (Figure 4).

## 3. Discussion

GSD is sporadic with no definite pattern of genetic inheritance [5]. The diagnosis of GSD relies on heightened clinical suspicion and diagnostic imaging, including X-rays, CT scans, MRIs, bone scintigraphy, and PET/CT scan (Table 1). Radiographs are the gold standard for detecting GSD, with characteristic findings of unilateral partial or total disappearance of bones, tapering of bony remnants, and absence of a sclerosing or osteoblastic reaction [7]. GSD is a diagnosis of exclusion after eliminating other conditions that may cause osteolysis (Table 2). The accurate diagnosis is challenging, and this condition is often misdiagnosed [12]. Heffez et al. have described eight inclusion criteria for the definitive diagnosis, including a (1) positive biopsy with the (2) absence of cellular atypia, (3) minimal or no osteoblastic response and absence of dystrophic calcification, (4) evidence of local, progressive osseous resorption, (5) a nonexpansive, nonulcerative lesion, (6) absence of visceral involvement, (7) an osteolytic radiographic pattern, and (8) a negative hereditary, metabolic, neoplastic, immunologic, or infectious etiology [15].

Treatment of GSD is symptom-based and is dependent on the location affected. Pharmacologic management consisting of nonsteroidal anti-inflammatory medications, vitamin D, calcium, corticosteroids, the anti-osteoclastic bisphosphonates, and alpha-2b interferon are usually successful in curtailing symptoms [4, 5, 7, 10, 11]. Radiotherapy may decelerate angiogenesis or halt the progression of osteolysis and is often utilized to decrease the size of the lesion [4–7]. Additionally, radiotherapy may accelerate sclerosis of the proliferating blood vessels and avert regrowth of these vessels [7]. Rapamycin, trametinib, and the chemotherapy agent bevacizumab have also been efficacious in GSD [6, 7]. For patients with a pathologic fracture or severe pain, surgical resection of the affected bone followed by prosthetic reconstruction may be performed [4–6]. Bone grafts are often not utilized as they undergo resorption [11].

Several cases have been reported in the literature of patients who underwent shoulder arthroplasty and developed radial nerve palsy postoperatively [16–21]. Sherfey and Edwards described a patient who underwent a humeral arthroplasty using a cemented fracture specific prosthesis [20]. On postoperative Day 1, the patient had evidence of a complete radial nerve palsy which was initially thought to be residual effects of the interscalene block. The patient continued to experience the radial nerve palsy six days postoperatively. Radiographs revealed cement extrusion from the shaft of the humerus near the distal tip of the implant. Following physical and occupational therapy for 6 months postoperatively, the patient attained resolution of the radial nerve palsy. These authors attributed the patient's radial nerve palsy to thermal injury of the radial nerve intraoperatively. In Vajapey et al.'s review of neurological complications in primary anatomic and reverse total shoulder arthroplasty, radial nerve palsy was most common due to an intraoperative periprosthetic fracture [21]. Several mechanisms have been reported, including inadvertent fracture of the humeral shaft after reaming and impaction of the humeral component, cement extravasation leading to thermal injury, and direct trauma from cerclage wire application in close proximity to the spiral groove [16–18]. An additional risk factor for radial nerve palsy is intraoperative positioning of the humerus [19]. Placing patients too supine on the operating table may lead to excessive extension of the shoulder; therefore, this should be avoided to minimize the likelihood of radial nerve palsy following shoulder arthroplasty.

An iatrogenic injury during the shoulder replacement surgery most likely caused the complication of radial nerve palsy in our case. Several causes may have resulted in the radial nerve injury in our case including stretch injury, direct injury to the radial nerve, or postoperative hematoma. We speculate that the most likely mechanism is stretch injury. Furthermore, radial nerve palsy may also occur in complicated cases where intraoperative length assessment is difficult to determine in shoulder arthroplasty. The mechanism of radial nerve palsy in this situation is most likely due to stretching of the radial nerve intraoperatively.

The patient in our report was 48 years old when he sustained an injury to the left shoulder which led to his first shoulder surgery. The surgeon who performed the revision of the reverse total shoulder arthroplasty 22 years after the first surgery did not ascertain the cause of the bone loss of the humerus and proceeded with the shoulder surgery. The surgery was likely to fail as more bone from the humeral shaft would be lost, and the prosthesis would likely come loose. The patient sustained an iatrogenic radial nerve injury during the shoulder replacement surgery causing wrist drop. The patient in our report was not formally diagnosed with GSD; however, the significant bone loss of the humerus noted on radiographical imaging and during the revision of the reverse total shoulder arthroplasty mimicked GSD. This case is valuable in that it brings awareness to similar situations that may be avoided in the future. This type of surgical revision usually involves shortening the limb length slightly. However, since a reverse shoulder prosthesis was needed, higher soft tissue tension was required.

## 4. Conclusion

GSD is marked by destruction of the osseous matrix and proliferation of vascular structures leading to bone absorption. The affected bone disintegrates and is replaced by vascular fibrous connective tissue. The condition is often missed unless the physician is familiar with this rare condition. The physician should also be aware of potential complications including spinal cord and nerve injury either from the osteolysis itself or from iatrogenic injuries during surgical treatment. The radial nerve may be injured during shoulder replacement with significant bone loss of the humerus resembling GSD. The mechanism of radial nerve palsy in our case is most likely due to stretching of the radial nerve during the shoulder replacement surgery. EDX and US studies are useful in confirming radial neuropathy that may occur following shoulder replacement surgery.

## Figures and Tables

**Table 1:** Diagnostic evaluation of Gorham–Stout disease.

Diagnostic tools	Characteristic findings
Laboratory	Alkaline phosphatase may be elevated

X-rays	Partial and total absence of junctional bone
	Lytic lesions in intramedullary and subcortical bone
	Absence of sclerosing or osteoblastic reactions
	Initial stage: Subcortical and intramedullary radiolucent foci
	Later stage: Osteolysis without osteosclerosis or periosteal reaction may appear as pathological fractures

CT scan	Bone loss and dissolution
	Extent of soft tissue involvement
	Diffuse atrophy of adjacent muscles due to bone resorption

MRI	Hypointense on T1-weighted images and hyperintense on T2-weighted images
	Bone loss and resorption
	Vascular and/or lymphatic vessels within bone in active osteolysis
	Diffuse atrophy of adjacent muscles

Bone scintigraphy	Increased uptake in areas with increased lymphatic and vascular proliferation
	Decreased uptake at osteolytic regions of vanished bone

PET/CT	Radioactive fluorine F 18-sodium fluoride (^18^F-NaF) identifies osteolytic foci

Histopathology	Early stage: Tissue from radiolucent defects: Nonspecific vascular proliferation mixed with fibrous connective tissue and chronic inflammatory cell infiltrate
	Later stage: Residual fibrous tissue replaces resorbed bone

**Table 2:** Differential diagnosis of Gorham–Stout disease.

Lymphangiomatosis
Multiple myeloma
Metastatic bone disease due to breast carcinoma
Hajdu–Cheney syndrome
Paget's disease
Rheumatoid arthritis
Fibrous dysplasia
Langerhans cell histiocytosis
Hyperparathyroidism
Aneurysmal bone cyst
Osteosarcoma
Winchester syndrome (Hardegger Type V classification)
Carpal tarsal osteolysis
Idiopathic multicentric osteolysis
Multicentric osteolysis with nephropathy
Eosinophilic granulomatosis
Syringomyelia

## Data Availability

The data that support the findings of this study are available from the corresponding author upon reasonable request.
